# Symptom-Specific Hospital Contacts in 12–18-Year-Olds Vaccinated against COVID-19: A Danish Register-Based Cohort Study

**DOI:** 10.3390/vaccines11061049

**Published:** 2023-05-31

**Authors:** Selina Kikkenborg Berg, Helle Wallach-Kildemoes, Line Ryberg Rasmussen, Ulrikka Nygaard, Nina Marie Birk, Henning Bundgaard, Annette Kjær Ersbøll, Lau Caspar Thygesen, Susanne Dam Nielsen, Anne Vinggaard Christensen

**Affiliations:** 1 Department of Cardiology, Rigshospitalet, Copenhagen University Hospital, Inge Lehmanns Vej 7, 2100 Copenhagen, Denmark; 2 Faculty of Health and Medical Sciences, University of Copenhagen, Blegdamsvej 3B, 2200 Copenhagen, Denmark; 3 Department of Paediatrics and Adolescents Medicine, Rigshospitalet, Copenhagen University Hospital, Blegdamsvej 9, 2100 Copenhagen, Denmark; 4 National Institute of Public Health, University of Southern Denmark, Studiestræde 6, 1455 Copenhagen, Denmark; 5 Department of Infectious Disease, Rigshospitalet, Copenhagen University Hospital, Blegdamsvej 9, 2100 Copenhagen, Denmark

**Keywords:** COVID-19, children and adolescents, vaccine, symptoms

## Abstract

In this register-based real-life cohort study, changes in symptom-specific hospital contacts among 12–18-year-olds following two doses of the BNT162b2 COVID-19 vaccine compared to unvaccinated peers were investigated. Using national register data, vaccinated and unvaccinated adolescents were sex and age-matched each week during the inclusion period from May to September 2021. Symptom-specific hospital contacts covering ICD-10 R diagnoses were assessed before first the vaccine dose and after the second vaccine dose. Taking previous rates of symptom-specific hospital contacts into account, differences between vaccinated and unvaccinated adolescents were found. For some hospital contacts, higher rates were seen among the vaccinated, and for others, higher rates were seen among the unvaccinated. Unspecific cognition symptoms may be important to monitor in vaccinated girls, and likewise for throat and chest pain in vaccinated boys within the first months post-vaccination. In perspective, symptom-specific hospital contacts after vaccination against COVID-19 must be assessed by taking the risk of infection and symptoms following COVID-19 infection into account.

## 1. Introduction

In May 2021, the U.S. Food and Drug Administration (FDA) and the European Medicines Agency (EMA) approved the first COVID-19 vaccine BNT162b2 (Pfizer-BioNTech) for emergency use in adolescents aged 12–15 years. The decision was based on a study by Frenck et al., which showed that BNT162b2 had an acceptable safety profile, a stronger immune response than in young adults, and was effective against SARS-CoV-2 [1]. The main side effects were pain at the injection site, fatigue, headache, chills, and muscle pain [1]. Further, very rare side effects have been reported to include perimyocarditis [2,3], Guillain–Barré syndrome [4], and multisystem inflammatory syndrome [5].

In healthy adolescents, SARS-CoV-2 infection is predominantly mild or asymptomatic [6], thus, the need for vaccination at an individual level is debated. However, severe illness may occur with increasing risk in adolescents with an underlying medical disease [7]. Additionally, SARS-CoV-2 infection is suggested to cause long-lasting symptoms in some adolescents [8,9]. Measures to control the pandemic caused educational disruption and social isolation in young people [10]. Hence, from a public health perspective, safe and effective vaccines were important for resocialization, education, and to contribute to herd immunity.

Vaccinated and unvaccinated adolescents are not necessarily comparable. Previous research has shown that they may differ in socioeconomic background and morbidity [11,12]. Therefore, when investigating health outcomes after vaccination against SARS-CoV-2 in a real-life setting, it is necessary to account for healthcare contacts prior to vaccination and thus, not look for absolute numbers of contacts.

The aim of the present study was to investigate changes in symptom-specific hospital contacts among adolescents following two doses of the BNT162b2 COVID-19 vaccine compared to unvaccinated peers.

## 2. Materials and Methods

### 2.1. Setting

Denmark began free of charge mass vaccination against SARS-CoV-2 for those aged 16–19 years and 12–15 years in May 2021 and July 2021, respectively.

Open public SARS-CoV-2 testing was available free of charge from late May 2020 until March 2023. Denmark was one of the countries testing the most, with a mean of almost ten reverse-transcriptase polymerase chain reaction (PCR) tests per person and as many antigen tests recorded in a national register.

During the pandemic, restrictions on healthcare contacts were implemented in Denmark to varying degrees following periods with high SARS-CoV-2 transmission.

### 2.2. Study Design and Population

This is a phase IV real-life cohort study with a follow-up in Danish national registers. The study population covers Danish adolescents aged 12–18 years with no prior positive SARS-CoV-2 test sampled from the LongCOVIDKidsDK study [8]. This included all children and adolescents aged 0–18 years who tested SARS-CoV-2-positive before July 2021. These were matched randomly at a 1:4 ratio by birthdate and sex with peers without positive tests (n = 323,234).

In this study, we included adolescents with and without first dose vaccination between 1 May and 30 September 2021.

Excluded were individuals who tested SARS-CoV-2-positive before the inclusion period; individuals with migration history within 12 months prior to May 2021 (securing at least 12 months of historic register information); individuals vaccinated against SARS-CoV-2 a vaccine other than BNT162b2; and individuals with less than three weeks between first and second vaccine dose or with missing information on parental education.

### 2.3. Study Size, Sampling Strategy, and Follow-Up Periods

For the first-dose vaccine population, each vaccinated person was randomly matched at a 1:1 ratio by sex and age group (12–15 and 16–18 years old) with an unvaccinated person each week during the inclusion period, accounting for the above-described variations.

The second-dose vaccine population was sampled from the first-dose vaccine population, applying an analogous sampling strategy. To be included as vaccinated, we required 3–7 weeks between the first and second vaccine doses, as recommended. Unvaccinated people were required not to be vaccinated against SARS-CoV-2 throughout the follow-up period. Two follow-up periods after the second dose index date were established: short (0–56 days) and long (57–182 days), excluding individuals dying or emigrating prior to day 56 after the index date in the long follow-up analyses.

While vaccinated individuals were assigned an index date corresponding to the vaccination date, the matched unvaccinated individuals were assigned an index date corresponding to the Wednesday in the sampling week. The age was defined as the age at a first-dose vaccine for the vaccinated individuals and as the age on the Wednesday of the first-dose vaccine sampling week for unvaccinated individuals. Applying sampling with replacement [13], each unvaccinated individual was matched with several vaccinated individuals and, thus, could be assigned several different index dates.

Both vaccinated and unvaccinated individuals were followed for the first registered positive test for SARS-CoV-2 infection, counting events and person-time at risk as uninfected until testing positive and, thereafter, as SARS-CoV-2-infected for the rest of the follow-up.

### 2.4. Data Sources

The following registers were used: the Danish Vaccination Register (DDV) [14], the Danish Microbiology Database (MiBa) [15], the Danish National Patient Register (DNPR) [16], and registers on sociodemographic information.

The study was permitted by the Danish Data Protection Agency (P-2021-195) and registered at clinicaltrials.gov (NCT04786353). Register data access was granted by The Danish Health Data Authority (FSEID 00005625). Ethics committee approvals are not required for surveys and register-based studies in Denmark.

### 2.5. Variables

Exposure was defined as vaccination with BNT162b2. Outcomes were secondary healthcare visits with information on a priori-selected ICD-10 R codes (Supplementary Table S1) as discharge diagnoses. The selected diagnoses covered symptoms and signs related to the circulatory and respiratory systems, digestive system and abdomen, skin, cognition and perception, and general symptoms, such as headache and fatigue. Selection of diagnoses was based on product resumes for the BNT162b2 vaccine, the websites of the Danish Medicines Agency, the Danish Medicine Information [17], the clinical decision support site UpToDate.com, along with a literature search on the suspected side effects in adolescents.

Dates of healthcare contacts were extracted from DNPR and calculated for each outcome measure person-time-at-risk and number of events 182 days prior to the index date and the relevant period after the index date. For the prior period, the first dose index date was applied. Events and person-year-at-risk (PYR) were added up over individual outcome measure observations, according to age and infection during follow-up.

A positive PCR test during follow-up was included as a time-dependent co-variable. Furthermore, sociodemographic information from 2019 for parents’ highest formal education (three levels), tertiles of family income, and maternal citizenship (Danish, other Western, non-Western), and prevalent health conditions covering binary somatic and psychiatric variables were included, as described in Table 1 and Supplementary Table S2.

### 2.6. Statistical Approach

In line with other Danish studies on SARS-CoV-2 infection [18,19], we applied prior event rate ratio (PERR) methodology [20,21]. The PERR method assumes that the ratio after the the prior period of ‘exposed to unexposed outcome event rate’ reflects the effect of the post-period ‘exposed to unexposed outcome event rate’ adjusted for measured and unmeasured time-independent confounders [20]. PERR estimates are presented with 95% confidence intervals (CIs). For PERR estimates >1, the pre- versus post-difference in outcome event rate among the vaccinated is higher than among the unvaccinated.

To enable adjustment for SARS-CoV-2 infection during the follow-up periods, multivariable Poisson regression analyses were used based on the PERR estimates on incidence rate ratios (IRR) for the before and after periods. As parental education was associated with vaccination status and most likely with healthcare contacts, we also adjusted for this time-independent confounder.

For each outcome measure and period of interest, PERR estimates were calculated, stratifying the analyses by sex. A minimum of five events in before and after periods were required in each stratum.

Bootstrapping [22] was used for calculating 95% CIs on PERR estimates, with 200 replicates of the original population randomly sampled with replacement for each outcome measure by strata for the post- and prior-period Poisson regression analyses.

Data management and statistical analyses were performed using StataCorp 2021 Stata Statistical Software: Release 17.

## 3. Results

### 3.1. Characteristics of the Study Population

**Figure 1:**
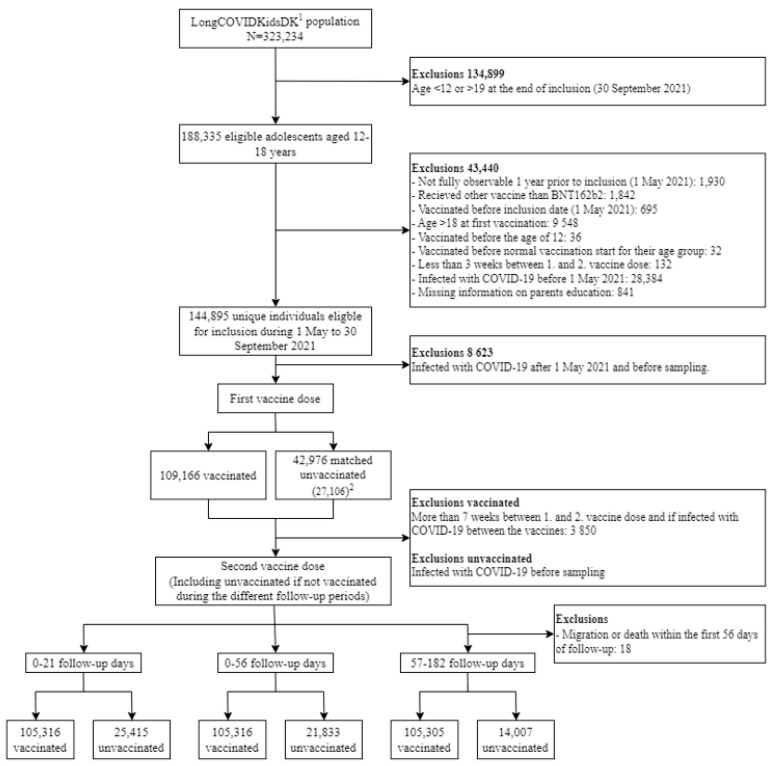
Flowchart describing the study population. (1) The LongCOVIDKidsDK population is described elsewhere [8]. (2) A total of 42,976 unique unvaccinated individuals were matched each week during the inclusion period with up to several vaccinated. Individuals once matched as unvaccinated may receive the first vaccine dose during the inclusion period and are then matched with those still unvaccinated. By the end of the inclusion period, 27,106 unique individuals were still unvaccinated.

The study population consisted of 105,316 people who were vaccinated and matched to 21,833 and 14,007 unvaccinated individuals throughout the short and long follow-up periods, respectively (Figure 1). The mean age was 15.6 years (SD 1.9) among the vaccinated and 15.4 years (2.1) among the unvaccinated, and 49% were girls in both groups. Proportions with somatic and psychiatric illnesses were similar. Parents of vaccinated adolescents had higher educational levels, higher incomes, and more had Danish citizenship compared to parents of unvaccinated adolescents (Table 1).

### 3.2. Symptom-Specific Hospital Contacts 0–56 Days after Second Vaccine Dose (Short Follow-Up)

**Figure 2:**
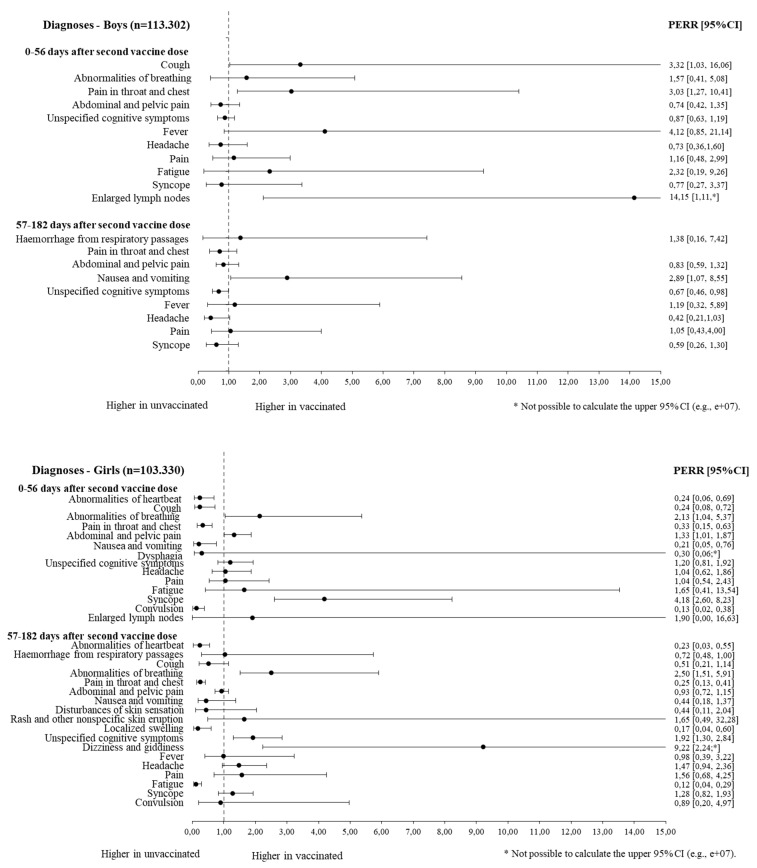
Forest plots of prior event rate ratios (PERR) for symptom-specific hospital contacts among vaccinated and unvaccinated girls and boys.

During the short follow-up period after the second vaccine, vaccinated girls had lower rates of hospital contacts with (ICD-10 R00) abnormal heartbeats (PERR 0.24 (0.06–0.69)), (ICD-10 R05) cough (0.24 (0.08–0.72)), (ICD-10 R07) pain in the throat and chest (0.33 (0.15–0.63)), (ICD-10 R11) nausea and vomiting (0.21 (0.05–0.76)), and (ICD-10 R56) convulsion (0.13 (0.02–0.38)) when taking into account their previous pattern of those contacts. In the same period, vaccinated girls had higher rates of (ICD-10 R06) abnormalities of breathing (2.13 (1.04–5.37)) and (ICD-10 R55) syncope (4.18 (2.60–8.23)). Vaccinated boys had higher rates of cough (3.32 (1.03–16.06)) and pain in the throat and chest (3.03 (1.27–10.41)) in the short follow-up period (Table 2, Figure 2).

### 3.3. Symptom-Specific Hospital Contacts 57–182 Days after Second Vaccine Dose (Long Follow-Up)

In the long follow-up period 57–182 days after the second vaccine, vaccinated girls had lower rates of abnormal heartbeat (0.23 (0.03–0.55)), pain in the throat and chest (0.25 (0.13–0.41)), (ICD-10 R22) localized swelling, mass and lump of the skin and subcutaneous tissue (0.17 (0.04–0.60)), and (ICD-10 R53) fatigue symptoms (0.12 (0.04–0.29)) compared to unvaccinated girls. During this period, vaccinated girls still had a higher rate of abnormalities of breathing (2.50 (1.51–5.91)), as well as a higher rate of (ICD-10 R41.8) other and unspecified symptoms and signs involving cognitive functions and awareness (1.92 (1.30–2.84)). Vaccinated boys had lower rates of other and unspecified symptoms and signs involving cognitive functions and awareness (0.67 (0.46–0.98)) and higher rates of nausea and vomiting (2.89 (1.07–8.55)) during the long follow-up period (Table 2, Figure 2).

Symptoms after the first vaccine dose and counts of diagnoses after both the first and second vaccine doses are presented in Supplementary Tables S3–S5.

## 4. Discussion

Differences in rates of selected symptom-specific hospital contacts were found between vaccinated and unvaccinated adolescents after vaccination against COVID-19. Vaccinated girls had lower rates of abnormal heartbeat, cough, pain in the throat and chest, nausea and vomiting, and convulsions up to 56 days after vaccination. By contrast, they had higher rates of abnormalities of breathing and syncope. Vaccinated boys had higher rates of cough and pain in the throat and chest during this period. After 57–182 days, vaccinated girls had lower rates of abnormal heartbeat, pain in the throat and chest, localized swelling, and fatigue. Further, they still had higher rates of abnormalities of breathing and unspecific cognition symptoms. Vaccinated boys had higher rates of nausea and vomiting and lower rates of unspecific cognition symptoms during long follow-ups after vaccination. The analyses accounted for previous patterns of hospital contacts related to the selected diagnoses six months prior to vaccination. Thus, the findings cannot be explained by differences in habitual healthcare use between vaccinated and unvaccinated adolescents.

This is the first study to explore symptom-specific hospital contacts in adolescents following vaccination with BNT162b2 using national real-life data. In addition to common expected vaccine side effects [1], previous research has mainly focused on safety in terms of side effects in the short period after vaccination and, on rare occasions, severe side effects [2,3,4,5] and efficacy [1,19] of the BNT162b2 vaccine.

A previous study from this cohort investigating self-reported symptoms in the first 14 days following BNT162b2 vaccination found that vaccinated adolescents reported more headaches, tiredness, and gastrointestinal symptoms compared to unvaccinated adolescents [23]. Similarly, in another American study using Vaccine Adverse Event Reporting System (VAERS) data, some of the most common self-reported symptoms were fatigue, headaches, chills, and pyrexia among vaccinated adolescents [24]. These were, however, self-reported symptoms that did not necessarily require contact with the hospital. Further, COVID-19 vaccination has been linked to menstrual disturbance, but a certain association has not been established [25].

The finding of fewer diagnoses of abnormal heartbeat, cough, pain in the throat and chest, nausea and vomiting and convulsions, and localized swelling and fatigue among vaccinated girls, and fewer unspecific cognition symptoms in vaccinated boys, are not easily interpreted. It is unlikely that the BNT162b2 vaccine prevents such hospital contacts not related to SARS-CoV-2.

The PERR estimates for the included symptoms are affected by the rates of hospital contacts among vaccinated and unvaccinated adolescents for each diagnosis in the period prior to vaccination. That is the strength of using this statistical method. For some of the included diagnoses, there seem to be changes in rates of hospital contacts before and after vaccination for both vaccinated and the unvaccinated adolescents. Among girls, for instance, the rate of pain in the throat and chest in the unvaccinated increased from 2.3 in the prior period to 10.9 per 1000 person-years at risk in the short follow-up period. Among vaccinated girls, the numbers were 3.4 and 5.9 per 1000 person-years at risk. Thus, the PERR estimate indicating a lower rate of pain in the throat and chest among vaccinated girls is explained by a larger increase in rates among unvaccinated compared to vaccinated girls. Similarly, among vaccinated boys, rates of pain in the throat and chest were 4.6 and 3.5 among unvaccinated and 2.6 and 5.3 per 1000 person-years at risk. Thus, the PERR estimate indicating an increased rate of pain in the throat and chest among vaccinated boys is explained by a larger increase in the rate among vaccinated than among unvaccinated boys.

The increased risk of hospital contacts due to unspecific cognitive symptoms during the long follow-up period among girls has not previously been reported. In the American study, VAERS data were reviewed from a total of 9246 reports from U.S. adolescents aged 12–17 years after receiving the BNT162b2 vaccine. The most common non-serious reports were for dizziness, in 1862 (21.2%) cases, and syncope, reported in 1228 (13.3%). This was, however, reported in the week following vaccination [26]. The number of unspecified symptoms and signs involving cognitive functions and awareness is relatively high. This may be interesting to explore in future studies, considering the introduction of an mRNA mass vaccination in adolescents.

There were differences between girls and boys in symptom-specific hospital contacts following vaccination. Sex differences in health are well-known and generally appear after puberty [27]. There were generally more hospital contacts among girls and more significant differences between vaccinated and unvaccinated girls. Sex differences in vaccine-related side effects following the mRNA COVID-19 vaccine have also been identified in adults [28]. In previous studies on this cohort, sex differences were also found in long-term symptoms following infection with SARS-CoV-2, with girls reporting more symptoms [8]. An increased risk of pain in the throat and chest among boys following vaccination with BNT162b2 has been reported previously and is in line with our findings. In the American VAERS data, the most commonly reported serious event was chest pain, reported in 56.4% of the 863 adolescents reporting serious events [26]. A study from Thailand found the most common cardiovascular effects after the second dose were tachycardia (7.6%), shortness of breath (6.6%), palpitations (4.3%), and chest pain (4.3%) [29]. Pain in the throat and chest can reflect non-harmful pain during an uncomplicated viral or bacterial infection. This may, however, also be a sign of perimyocarditis. From June 2021, reports began to surface about cases of perimyocarditis after receiving, especially, the second dose of the BNT162b2 vaccine, which were primarily reported in young males [2,3]. In a Danish population-based study, the incidence of perimyocarditis among males and females aged 12–17 years was 1 in 10,000 males and 1 in 63,000 females, respectively. The equivalent incidence of multisystem inflammatory syndrome-related myocarditis after infection with SARS-CoV-2 was 1 in 2800 males and 1 in 5300 females [30].

A major strength of this study is the use of real-life data from the Danish nationwide health registries allowing us to link and follow the cohort of Danish adolescents before and after the index date. Another major strength is the weekly sampling and matching strategy to reflect the changing SARS-CoV-2 load and Danish pandemic policy. Using ICD-10 R-diagnoses as an outcome measure enabled us to capture registered complaints and symptoms.

In line with previous Danish studies [18,19], PERR methodology was applied to adjust for any non-time-dependent confounders. Nonetheless, we applied Poisson regression models to account for SARS-CoV-2 infection during follow-up. As vaccination against SARS-CoV-2 was offered free of charge, accepting the vaccine was, thus, dependent on other factors, such as general health beliefs [31]. As sociodemographic variables are strongly associated with vaccination, parental education was included as a time-independent confounder.

External validity is supported by Denmark adhering to international guidelines for the vaccination of adolescents. Comprehensive characteristics of the population are presented to allow for comparison between groups. Ecological validity is high, as we report from a real-life setting.

The study also has limitations. The study population is sampled from a study including infected adolescents and matched controls [8]. It is unlikely that this sub-population per se has introduced important selection bias in the present study exploring healthcare use among those without a positive test after subsequent vaccination.

As censoring when the unvaccinated got vaccinated would introduce bias [21], the unvaccinated had to remain unvaccinated throughout follow-up. The reference group of the unvaccinated most likely covers adolescents (or their parents) with a negative stance on the vaccine, which is unlikely to be related to their health conditions. Hence, it is unlikely that important selection bias has been introduced by looking into the future regarding vaccination.

Applying a 1:1 sampling ratio with replacement [13] implied that most unvaccinated contributed as a reference with several index dates. As Poisson analyses are based on events and person-time-at-risk (cumulative outcome rates), the ‘re-use’ of unvaccinated individuals should not, per se, introduce bias.

Some of the bootstrapped samples applied to estimate a 95% CI on PERR estimates resulted in non-convergence of the Poisson regression model due to few events, implying a ‘non-estimable’ 95% CI. The low number of some diagnoses and few events per 1000 PYR need to be considered when interpreting the PERR results in a clinical setting.

To conclude, in the time following the second dose of the vaccine against COVID-19, differences were found in symptom-specific hospital contacts between vaccinated and unvaccinated adolescents when taking previous patterns of hospital contacts in the groups into account. Unspecific cognition symptoms may be important to monitor in girls, and likewise for throat and chest pain in boys within the first months post-vaccination. In perspective, symptom-specific hospital contacts after vaccination against COVID-19 must be assessed by taking the risk of infection and symptoms following COVID-19 infection into account.

## Figures and Tables

**Table 1:** Characteristics of adolescents vaccinated against COVID-19 and matched unvaccinated indviduals. The first dose was during the inclusion period of May–September and the second dose was 3–7 weeks later.

	Second Dose of the Vaccine ^a^		
	Vaccinated	Unvaccinated According to Follow-Up	
		0–56 Days	57–182 Days
**All n (%) ^b^**	**(n = 105,316)**	**(n = 105,316)**	**(n = 105,316)**
**Unique individuals, n (%) ^c^**	105,316 (100.0%)	21,833 (20.7%)	14,007 (13.3%)
**Sex (girl, %)**	51,665 (49.1%)	51,665 (49.1%)	51,665 (49.1%)
**Age (mean, SD)**	15.5 (1.9)	15.4 (2.1)	15.4 (2.1)
**12–15 years (%)**	46,733 (44.4%)	46,733 (44.4%)	46,733 (44.4%)
**16–18 years (%)**	58,583 (55.6%)	58,583 (55.6%)	58,583 (55.6%)
**Prevalent health condition, n/yes (%) ^d^**			
**Any of the listed somatic diseases**	13,971 (13.3%)	12,324 (11.7%)	12,197 (11.6%)
**Any registered psychiatric diagnosis**	8377 (8.0%)	10,284 (9.8%)	10,468 (9.9%)
**Parents’ socioeconomic position**			
**Parents’ highest formal education, n (%)**			
**Basic education**	4737 (4.5%)	15,230 (14.5%)	17,631 (16.7%)
**High school and vocational training**	43,245 (41.1%)	50,777 (48.2%)	52,260 (49.6%)
**Higher education**	57,334 (54.4%)	39,309 (37.3%)	35,425 (33.6%)
**Annual family income, n (%)**			
**Low (1. tertile)**	27,615 (26.2%)	54,884 (52.1%)	59,961 (56.9%)
**Middle (2. tertile)**	33,168 (31.5%)	29,734 (28.2%)	28,450 (27.0%)
**High (3. tertile)**	43,984 (41.8%)	19,715 (18.7%)	15,901 (15.1%)
**Maternal citizenship, n (%)**			
**Danish**	97,754 (92.8%)	82,037 (77.9%)	78,905 (74.9%)
**Other Western countries**	3089 (2.9%)	5922 (5.6%)	6778 (6.4%)
**Non-Western countries**	4277 (4.1%)	16,684 (15.8%)	18,928 (18.0%)
**Unknown**	196 (0.2%)	673 (0.6%)	705 (0.7%)

^a^ Applying matching with replacement, the unvaccinated were matched to second dose vaccinated according to sex and age group. Merely individuals not receiving first dose of the vaccine during the relevant follow-up periods were included. ^b^ Matched the number of index date records applied in the analyses. Unvaccinated individuals could be assigned several index dates. Index date among the vaccinated = date of two. Dose vaccination. Prevalent health conditions and parental socioeconomic position are based on these matched number of index date records. ^c^ Number of unique individuals who applied for the matching. By the end of the inclusion period, 27,106 individuals had not received the first vaccine dose. ^d^ Somatic or psychiatric disorders registered prior to first dose vaccine index date: for details, see Supplementary Table S1.

**Table 2:** PERR ^a^ and the number of symptoms (ICD-10 R-codes) ^b^ per 1000 person-years at risk (rates) 6 months before and two time periods after the second vaccine dose (index date) among vaccinated and matched unvaccinated adolescents aged 12–18 years.

		Girls						Boys					
		Unvaccinated (Rate)		Vaccinated (Rate)				Unvaccinated (Rate)		Vaccinated (Rate)			
		Before	After	Before	After	PERR Crude	PERR (95% CI) ^c^	Before	After	Before	After	PERR Crude	PERR (95% CI) ^c^
	***Short (0–56 days)***												
Circulatory and respiratory symptoms	R00 Abnormal heartbeat	1.4	5.9	2.3	1.9	0.20	0.24 (0.06–0.69)	-	-	-	-	-	-
	R05 Cough	0.9	4.5	2.1	2.7	0.26	0.24 (0.08–0.72)	1.8	1.2	0.7	1.5	2.98	3.32 (1.03–16.06)
	R06 Abnormalities of breathing	8.0	2.9	5.4	4.7	2.37	2.13 (1.04–5.37)	2.5	3.8	2.0	3.2	1.03	1.57 (0.41–5.08)
	R07 Pain in the throat and chest	2.3	10.9	3.4	5.9	0.37	0.33 (0.15–0.63)	4.6	3.5	2.6	5.3	2.70	3.03 (1.27–10.41)
Digestive system and abdomen	R10 Abdominal and pelvic pain	54.88	35.52	44.17	37.73	1.32	1.33 (1.01–1.87)	14.27	18.09	15.38	15.12	0.78	0.74 (0.42–1.35)
	R11 Nausea and vomiting	1.87	5.80	4.37	4.29	0.32	0.21 (0.05–0.76)	-	-	-	-	-	-
	R13 Dysphagia	0.59	0.76	1.66	1.14	0.54	0.30 (0.06-#)	-	-	-	-	-	-
Cognition, perception, emotional state, and behavior	R41.8 Other and unspecified symptoms and signs involving cognitive functions and awareness	42.1	40.8	23.8	26.5	1.15	1.20 (0.81–1.92)	37.4	38.4	22.1	20.8	0.92	0.87 (0.63–1.19)
General symptoms and signs	R50 Fever	-	-	-	-	-	-	0.6	0.6	0.5	1.8	3.68	4.12 (0.85–21.14)
	R51 Headache	18.0	15.1	15.7	13.4	1.01	1.04 (0.62–1.86)	6.1	9.7	5.9	6.0	0.63	0.73 (0.36–1.60)
	R52 Pain	5.2	5.4	6.2	5.4	0.84	1.04 (0.54–2.43)	3.7	2.8	2.9	2.3	1.03	1.16 (0.48–2.99)
	R53 Fatigue	2.8	1.9	3.0	2.7	1.30	1.65 (0.41–13.54)	0.7	1.0	2.1	3.5	1.27	2.32 (0.19–9.26)
	R55 Syncope	12.6	4.7	7.0	10.7	4.12	4.18 (2.60–8.23)	1.8	2.7	3.2	4.4	0.93	0.77 (0.27–3.37)
	R56 Convulsion	0.5	2.7	1.7	1.0	0.12	0.13 (0.02–0.38)	-	-	-	-	-	-
	R59 Enlarged lymph nodes	0.7	1.0	0.6	1.3	1.37	1.90 (0.00–16.63)	3.0	0.7	0.3	1.3	18.18	14.15 (2.11-#)
	***Long (57–182 days)***												
Circulatory and respiratory symptoms	R00 Abnormal heartbeat	0.5	3.5	2.3	3.1	0.22	0.23 (0.03–0.55)	-	-	-	-	-	-
	R04 Hemorrhage from respiratory passages	1.6	1.3	0.5	1.1	2.60	1.03 (0.29–5.74)	1.0	1.2	0.9	1.1	0.96	1.38 (0.16–7.42)
	R05 Cough	1.1	3.5	2.1	3.1	0.46	0.51 (0.21–1.14)	-	-	-	-	-	-
	R06 Abnormalities of breathing	10.7	3.8	5.1	4.4	2.46	2.50 (1.51–5.91)	-	-	-	-	-	-
	R07 Pain in the throat and chest	2.9	14.0	3.3	4.9	0.30	0.25 (0.13–0.41)	2.7	8.5	2.4	3.9	0.51	0.71 (0.38–1.26)
Digestive system and abdomen	R10 Abdominal and pelvic pain	61.14	69.71	42.25	42.43	0.88	0.93 (0.72–1.15)	16.52	23.37	15.53	15.40	0.70	0.83 (0.59–1.32)
	R11 Nausea and vomiting	5.04	10.12	4.26	4.86	0.57	0.44 (0.18–1.37)	4.73	2.64	2.06	3.18	2.77	2.89 (1.07–8.55)
Skin and subcutaneous tissue	R20 Disturbances of skin sensation	0.53	0.89	0.70	0.73	0.61	0.44 (0.11–2.04)	-	-	-	-	-	-
	R21 Rash and other nonspecific skin eruptions	2.71	1.45	1.03	1.17	2.12	1.65 (0.49–32.28)	-	-	-	-	-	-
	R22 Localized swelling, mass, and lump of skin and subcutaneous tissue	0.53	2.74	0.70	0.61	0.17	0.17 (0.04–0.60)	-	-	-	-	-	-
Cognition, perception, emotional state, and behavior	R41.8 Other and unspecified symptoms and signs involving cognitive functions and awareness	30.7	31.4	19.7	31.9	1.59	1.92 (1.30–2.84)	38.7	53.6	21.4	22.5	0.76	0.67 (0.46–0.98)
	R42 Dizziness and giddiness	5.0	0.6	3.4	3.3	7.79	9.22 (2.24-#)	-	-	-	-	-	-
General symptoms and signs	R50 Fever	4.6	3.5	3.0	2.0	0.87	0.98 (0.39–3.22)	1.0	0.8	2.2	2.3	1.47	1.19 (0.32–5.89)
	R51 Headache	26.8	15.8	15.2	12.9	1.43	1.47 (0.94–2.36)	6.0	14.8	6.3	6.5	0.42	0.42 (0.21–1.03)
	R52 Pain	7.4	5.3	5.5	4.8	1.23	1.56 (0.68–4.25)	2.8	2.4	3.1	2.8	1.04	1.05 (0.43–4.00)
	R53 Fatigue	0.9	6.0	1.7	1.3	0.11	0.12 (0.04–0.29)	-	-	-	-	-	-
	R55 Syncope	11.6	12.6	7.1	8.3	1.07	1.28 (0.82–1.93)	2.1	6.2	3.1	4.7	0.52	0.59 (0.26–1.30)
	R56 Convulsion	0.5	0.6	1.4	1.7	0.90	0.89 (0.20–4.97)	-	-	-	-	-	-

^a^ PERR (prior event rate ratio) = the ratio after (vaccinated rate after/unvaccinated rate after) divided by the ratio before (vaccinated rate before/unvaccinated rate before). ^b^ For the number of events for each symptom, see Supplementary Table S5. ^c^ By means of Poisson regression models, the ratio before and after is adjusted for time with SARS-CoV-2 infection in the follow-up period, highest attained parental education, and age group (12–15 years and 16–18 years during the inclusion period). There was a 95% confidence interval (CI) of the PERR estimate through bootstrapping with 200 replicates. # It is not possible to calculate the upper 95% CI.

## Data Availability

Deidentified individual participant data cannot be shared according to Danish legislation on data protection. However, all registers used in the current study are public, and access can be applied for through The Danish Health Data Authority.

## Supplementary Materials

The following supporting information can be downloaded at https://www.mdpi.com/article/10.3390/vaccines11061049/s1, Supplementary Table S1: Selected ICD-10 R codes as outcomes; Supplementary Table S2. Joint binary register-based measures for prevalent somatic and psychiatric disorders prior to first dose vaccine index date, applying hospital diagnosis (ICD 10 codes) and eventually medicine proxies (ATC-codes) with a specified look-back period; Supplementary Table S3. Characteristics of adolescents vaccinated against COVID-19 and matched with the unvaccinated: first dose during May–September and second dose 3–7 weeks later; Supplementary Table S4. PERR and the number of symptoms (ICD-10 R-codes) per 1000 person-years at risk (rates) 6 months before and 21 days after the first vaccine dose (index date) among vaccinated and matched unvaccinated adolescents aged 12–18 years; Supplementary Table S5. Symptoms for the first and second vaccine dose: events 6 months before and three time periods after vaccination (index date) among vaccinated and matched unvaccinated adolescents aged 12–18 years; stratified based on age groups.
