# Supplementary material for: Symptom-Specific Hospital Contacts in 12–18-Year-Olds Vaccinated against COVID-19: A Danish Register-Based Cohort Study
Source: Vaccines (Basel). 2023 May 31;11(6):1049. doi: 10.3390/vaccines11061049 (PMC10301149; doi:10.3390/vaccines11061049)
Supplement: Supplementary file 1 [file vaccines-11-01049-s001.zip › Supplementary_data.docx]

**Supplemental Information**

Supplement to: Berg SK, Wallach-Kildemoes H, Rasmussen LR, et al. Symptom-specific hospital contacts in 12-18-year-olds vaccinated against COVID-19: A Danish register-based cohort study

[Investigators 2](#_Toc131419787)

[Supplemental Table 1. Selected ICD-10 R codes as outcomes. 3](#_Toc131419788)

[Supplemental Table 2. Joint binary register-based measures for prevalent somatic and psychiatric disorders prior to first dose vaccine index-date, applying hospital diagnosis (ICD 10 codes) and eventually medicine proxies (ATC-codes) with specified look back period. 4](#_Toc131419789)

[Supplemental Table 3. Characteristics of adolescents vaccinated against COVID-19 and matched unvaccinated: First dose during May-September and second dose 3-7 weeks later. 5](#_Toc131419790)

[Supplemental Table 4. PERR and number of symptoms (ICD-10 R-codes) per 1000 person years at risk (rates) 6 months before and 21 days after the first vaccine dose (index-date) among vaccinated and matched unvaccinated adolescents aged 12-18 years. 6](#_Toc131419791)

[Supplemental Table 5. Symptoms for first and second vaccine dose: Events 6 months before and three time periods after vaccination (index-date) among vaccinated and matched unvaccinated adolescents aged 12-18 years, stratified on age-groups. 7](#_Toc131419792)

#

# Investigators

Selina Kikkenborg Berg, Professor^a,b^, Helle Wallach-Kildemoes, PhD^a^, Line Ryberg Rasmussen, Msc^a^, Ulrikka Nygaard, Associate Professor^b,c^, Nina Marie Birk, PhD^a^, Henning Bundgaard, Professor^a,b^, Annette Kjær Ersbøll, Professor^d^, Lau Casper Thygesen, Professor^d^, Susanne Dam Nielsen, Professor^b,e^, Anne Vinggaard Christensen, Post doc^a^

^a^Department of Cardiology, Rigshospitalet, Copenhagen University Hospital, Inge Lehmanns Vej 7, 2100 Copenhagen, Denmark

^b^Faculty of Health and Medical Sciences, University of Copenhagen, Blegdamsvej 3B, 2200 Copenhagen N, Denmark

^c^Department of Paediatrics and Adolescents Medicine, Rigshospitalet, Copenhagen University Hospital, Blegdamsvej 9, 2100 Copenhagen, Denmark

^d^National Institute of Public Health, University of Southern Denmark, Studiestræde 6, 1455 Copenhagen, Denmark

^e^Department of Infectious Disease, Rigshospitalet, Copenhagen University Hospital, Blegdamsvej 9, 2100 Copenhagen, Denmark

**Corresponding author:**

Professor Selina Kikkenborg Berg
Rigshospitalet

The Heart Centre, Unit 2151
Blegdamsvej 9

2100 Copenhagen
Denmark

Phone: +45 35459526

E-mail: [selina@rh.dk](mailto:selina@rh.dk)

# Supplemental Table 1. Selected ICD-10 R codes as outcomes.

| **R codes categories** | **Specific R codes** |
| --- | --- |
| Circulatory and respiratory symptoms | R00 Abnormalities of heartbeat |
|  | R04 Haemorrhage from respiratory passages |
|  | R05 Cough |
|  | R06 Abnormalities of breathing |
|  | R07 Pain in throat and chest |
|  | R09 Other symptoms and signs involving the circulatory and respiratory systems |
| Digestive system and abdomen | R10 Abdominal and pelvic pain |
|  | R11 Nausea and vomiting |
|  | R13 Dysphagia |
| Skin and subcutaneous tissue | R20 Disturbances of skin sensation |
|  | R21 Rash and other nonspecific skin eruption |
|  | R22 Localized swelling, mass and lump of skin and subcutaneous tissue |
| Cognition, perception, emotional state, and behavior | R41.8 Other and unspecified symptoms and signs involving cognitive functions and awareness |
|  | R42 Dizziness and giddiness |
|  | R43 Disturbances of smell and taste |
| General symptoms and signs | R50 Fever of other and unknown origin |
|  | R51 Headache |
|  | R52 Pain, not elsewhere classified |
|  | R53 Malaise and fatigue |
|  | R55 Syncope and collapse |
|  | R56 Convulsions, not elsewhere classified |
|  | R59 Enlarged lymph nodes |

Supplemental Table 2. Joint binary register-based measures for prevalent somatic and psychiatric disorders prior to first dose vaccine index-date, applying hospital diagnosis (ICD 10 codes) and eventually medicine proxies (ATC-codes) with specified look back period.

| **Disorder categories** | **Register-based disorder defintions^a^:**  **Prior diagnosis (ICD 10) / filled of prescription drug (ATC)** | **Look back period^b^** |
| --- | --- | --- |
| Asthma^c^ | **ICD10** |  |
|  | J45-J46 | Full |
|  | **ATC** |  |
|  | Bronchodilating agents(comb): R03BA; R03AK; R03AL08; R03AL09; R03AL11-12; R03AC12- R03AC19; R03BB01; | Medium |
|  | Leukotriene receptor antagonist: R03DC03 | Medium |
| Other respiratory disorders^c^ | **ICD10** |  |
|  | E84; J41-44; J47; J84; J96.1; P27; | Full |
|  | J40 | Short |
| Cardiovascular disorders^c^ | **ICD10** |  |
|  | I05-I09; I27; I34-39; I41-I47; I50-I52 | Full |
|  | I01; I31; I33; I40 | Medium |
| Renal disorders incl. dialysis^c^ | **ICD10:** |  |
|  | N03; N04-5; N07-8; N18-19; N25-N27; Y84.1 | Full |
|  | N00-1; N17 | Medium |
| Diabetes mellitus I or II^c^ | **ICD10:** |  |
|  | E10-E11; Z96.4 | Full |
|  | **ATC:** |  |
|  | A10A | Medium |
| Endocrine disorders. excl. diabetes^c^ | **ICD10** |  |
|  | E03.3; E03.5-9; E05-06; E27.1; E27.3-4 | Full |
|  | E27.2 | Short |
| Hematologic disorders^c^ | **ICD10** |  |
|  | D59.0-1; D69.0; D69.3; D86 | Full |
| Dermatologic diseases^c^ | **ICD10** |  |
|  | L10; L12; L13.0 | Medium |
|  | L20; L40; L63; L80 | Full |
| Rheumatological disorders^c^ | **ICD10** |  |
|  | M05-M09; M30-36; M45; M60 (excl. M60.0) | Full |
| Neuromuscular disorders^c^ | **ICD10** |  |
|  | G35-37; G70-71; G80; G82 | Full |
|  | G61; | Medium |
| Gastrointestinal disorders incl. cirrhosis (any cause)^c^ | **ICD10** |  |
|  | K50-51; K70.2-3; K71.7; K74; K76.1; K78.8; K900; | Full |
|  | K35 | Short |
| Congenital malformations and chromosomal abnormalities^c^ | **ICD10** |  |
|  | Q20-28; Q31-34; Q90-95 | Full |
| Malignancy^c^ | **ICD10** |  |
|  | C00-97 | Full |
|  | **ATC** |  |
|  | L01 | Full |
| Organ transplantation or immunodeficiency^c^ | **ICD 10** |  |
|  | Organ transplantation: Z94 excl. Z94.5. Z94.7 | Full |
|  | Immunodeficiency: D70-72; D730; D80-84 | Full |
|  | **ATC** |  |
|  | L04 | Full |
| Psychiatric disorders^d^ | ICD10 |  |
|  | F (any psychiatric primary diagnoses) | Full |

^a^Individuals with a list of disorders within a look back period prior to survey completion date were identified, applying individual-level register information, corresponding to primary discharge diagnoses (ICD-10 codes) and/or prescription drug use disease proxies (ATC codes). The list includes somatic health conditions in adolescents conferring an increased risk of SARS-CoV-2 infection^46^ along with any psychiatric discharge disorders.
^b^Looking back periods from the index-date: Full (ICD: full historic); Medium (ICD: 1 year; ATC: 6 months); Short (ICD: 3 months; ATC: 2 month); Very short: 14 days (ICD & ATC).
^c^A joint binary measure for prevalent somatic disorder: Yes (Yes in any of the listed prevalent disorders) No (No as to all included prevalent disorders).
^d^A joint measure for prevalent psychiatric disorder.

# Supplemental Table 3. Characteristics of adolescents vaccinated against COVID-19 and matched unvaccinated: First dose during May-September and second dose 3-7 weeks later.

| Category | First dose vaccine^a^ | | Second dose vaccine^b^ | | | |
| --- | --- | --- | --- | --- | --- | --- |
|  | **Vaccinated** | **Unvaccinated** | **Vaccinated** | **Unvaccinated according to follow-up** | | |
|  |  |  |  | **0-21 days** | **0-56 days** | **57-182 days** |
| All n (%)^c^ | (n=109,166) | (n=109,166) | (n=105,316) | (n=105,316) | (n=105,316) | (n=105,316) |
| Unique individuals, n (%)^d^ | 109,166 (100.0%) | 27,106 (39.4%) | 105,316 (100.0%) | 25,415 (24.1%) | 21,833 (20.7%) | 14,007 (13.3%) |
| Sex (Girl, %) | 53,423 (48.9%) | 53,423 (48.9%) | 51,665 (49.1%) | 51,665 (49.1%) | 51,665 (49.1%) | 51,665 (49.1%) |
| Age (mean, SD) | 15.6 (1.9) | 15.4 (2.1) | 15.5 (1.9) | 15.4 (2.1) | 15.4 (2.1) | 15.4 (2.1) |
| 12 - 15 years (%) | 47,719 (43.7%) | 47,719 (43.7%) | 46,733 (44.4%) | 46,733 (44.4%) | 46,733 (44.4%) | 46,733 (44.4%) |
| 16 - 18 years (%) | 61,447 (56.3%) | 61,447 (56.3%) | 58,583 (55.6%) | 58,583 (55.6%) | 58,583 (55.6%) | 58,583 (55.6%) |
| Prevalent health condition, n/yes (%)^e^ |  |  |  |  |  |  |
| Any of listed somatic diseases | 14,456 (13.2%) | 12,961 (11.9%) | 13,971 (13.3%) | 12,220 (11.6%) | 12,324 (11.7%) | 12,197 (11.6%) |
| Any registered psychiatric diagnosis | 8780 (8.0%) | 10,054 (9.2%) | 8377 (8.0%) | 9966 (9.5%) | 10,284 (9.8%) | 10,468 (9.9%) |
| Parental socio-economic position |  |  |  |  |  |  |
| Highest formal parental education, n (%) |  |  |  |  |  |  |
| Basic education | 5087 (4.7%) | 13,098 (12.0%) | 4737 (4.5%) | 14,890 (14.1%) | 15,230 (14.5%) | 17,631 (16.7%) |
| High school and vocational training | 44,777 (41.0%) | 50,522 (46.3%) | 43,245 (41.1%) | 49,746 (47.2%) | 50,777 (48.2%) | 52,260 (49.6%) |
| Higher education | 59,302 (54.3%) | 45,546 (41.7%) | 57,334 (54.4%) | 40,680 (38.6%) | 39,309 (37.3%) | 35,425 (33.6%) |
| Annual family income, n (%) |  |  |  |  |  |  |
| Low (1. tertile) | 29,055 (26.6%) | 50,078 (45.9%) | 27,615 (26.2%) | 53,858 (51.1%) | 54,884 (52.1%) | 59,961 (56.9%) |
| Middle (2. tertile) | 34,172 (31.3%) | 31,824 (29.2%) | 33,168 (31.5%) | 29,595 (28.1%) | 29,734 (28.2%) | 28,450 (27.0%) |
| High (3. tertile) | 45,359 (41.6%) | 26,296 (24.1%) | 43,984 (41.8%) | 20,857 (19.8%) | 19,715 (18.7%) | 15,901 (15.1%) |
| Maternal citizenship, n (%) |  |  |  |  |  |  |
| Danish | 101,063 (92.6%) | 89,218 (81.7%) | 97,754 (92.8%) | 82,554 (78.4%) | 82,037 (77.9%) | 78,905 (74.9%) |
| Other Western countries | 3261 (3.0%) | 5194 (4.8%) | 3089 (2.9%) | 5685 (5.4%) | 5922 (5.6%) | 6778 (6.4%) |
| Non-western countries | 4621 (4.2%) | 14,160 (13.0%) | 4277 (4.1%) | 16,409 (15.6%) | 16,684 (15.8%) | 18,928 (18.0%) |
| Unknown | 221 (0.2%) | 594 (0.5%) | 196 (0.2%) | 668 (0.6%) | 673 (0.6%) | 705 (0.7%) |

^a^Applying matching with replacement, unvaccinated were each week during the inclusion period (May-September 2021) matched to first dose vaccinated according to sex and age-group. Hence, unvaccinated may receive the vaccine during the inclusion period and be matched with an individual still unvaccinated. By the end of the inclusion period 27,058 unique individuals were still unvaccinated.

^b^Applying matching with replacement, unvaccinated were each week matched to second dose vaccinated according to sex and age-group. Merely individuals not receiving first dose vaccine during the respective follow-up periods were included.
^c^Matched number of index-date records applied in the analyses. Individuals still unvaccinated may have been assigned several index-dates. Index-date among vaccinated = date of vaccination. Prevalent health conditions and parental socio-economic position are based on these matched number of index-date records.

^d^Number unique individual applied for the matching. By the end of the inclusion period 27,058 individuals had not received first dose vaccine.
^e^Somatic or psychiatric disorders registered prior to first dose vaccine index-date: For details see Supplemental Table 2.

# Supplemental Table 4. PERR^a^ and number of symptoms (ICD-10 R-codes)^b^ per 1000 person years at risk (rates) 6 months before and 21 days after the first vaccine dose (index-date) among vaccinated and matched unvaccinated adolescents aged 12-18 years.

|  | **Girls** | | | | | | **Boys** | | | | | |
| --- | --- | --- | --- | --- | --- | --- | --- | --- | --- | --- | --- | --- |
|  | **Unvaccinated (Rate)** | | **Vaccinated**  **(Rate)** | |  | | **Unvaccinated (Rate)** | | **Vaccinated**  **(Rate)** | |  | |
|  | **before** | **after** | **before** | **after** | **PERR crude** | **PERR (95% CI)^c^** | **before** | **after** | **before** | **after** | **PERR crude** | **PERR (95% CI)^c^** |
| **1. Vaccine** |  |  |  |  |  |  |  |  |  |  |  |  |
| ***(0-21 days)*** |  |  |  |  |  |  |  |  |  |  |  |  |
| R00 Abnormalities of heartbeat | 1.67 | 1.95 | 2.24 | 2.93 | 1.12 | 1.23 (0.34-#) | - | - | - | - | - | - |
| R06 Abnormalities of breathing | 5.93 | 11.06 | 5.31 | 4.23 | 0.43 | 0.35 (0.14-1.26) | - | - | - | - | - | - |
| R07 Pain in throat and chest | 3.37 | 9.76 | 3.48 | 4.23 | 0.42 | 0.46 (0.18-1.05) | 3.53 | 2.49 | 2.62 | 6.86 | 3.71 | 4.70 (1.68-21.33) |
| R10 Abdominal and pelvic pain | 47.41 | 42.95 | 44.54 | 38.08 | 0.94 | 0.91 (0.65-1.42) | 15.84 | 18.40 | 15.27 | 14.66 | 0.83 | 0.90 (0.46-1.84) |
| R21 Rash and other nonspecific skin eruption | - | - | - | - | - | - | 0.71 | 2.18 | 0.75 | 1.56 | 0.68 | 0.63 (0.11-4.08) |
| R41.8 Other and unspecified symptoms and signs involving cognitive functions and awareness | 39.01 | 20.82 | 23.48 | 21.81 | 1.74 | 1.72 (1.10-2.96) | 33.05 | 41.17 | 23.22 | 19.34 | 0.67 | 0.61 (0.32-1.45) |
| R42 Dizziness and giddiness | 3.04 | 3.25 | 3.44 | 2.60 | 0.71 | 0.86 (0.11-#) | - | - | - | - | - | - |

^a^PERR (Prior event rate ratio) = Ratio after (Vaccinated Rate after/Unvaccinated Rate after) divided by the Ratio before (Vaccinated Rate before/Unvaccinated Rate before).
^b^For the number of events for each symptom see Supplemental Table 5.
^v^By means of Poisson regression models, the ratio before and after is adjusted for time with SARS-CoV-2 infection in the follow-up period, highest attained parental education and age group (12-15 years and 16-18 years during inclusion period). 95% confidence interval (CI) of the PERR estimate through Bootstrapping with 200 replicates.
^#^ Not possible to calculate the upper 95% CI (e.g., e+07).

# Supplemental Table 5. Symptoms for first and second vaccine dose: Events 6 months before and three time periods^a^ after vaccination (index-date) among vaccinated and matched unvaccinated adolescents aged 12-18 years, stratified on age-groups.

| **Diagnoses by length of follow-up period** | | **Non-vaccinated: Events (before/after)** | | | | **Vaccinated: Events (before/after)** | | | |
| --- | --- | --- | --- | --- | --- | --- | --- | --- | --- |
|  |  | **Girls** | | **Boys** | | **Girls** | | **Boys** | |
|  |  | **12-18** | | **12-18** | | **12-18** | | **12-18** | |
|  |  | *before* | *after* | *before* | *after* | *before* | *after* | *before* | *after* |
|  | **1. vaccine** |  |  |  |  |  |  |  |  |
|  | ***Very-short (0-21 days)*** |  |  |  |  |  |  |  |  |
| Circulatory and respiratory symptoms | R00 Abnormalities of heartbeat | 45 | 6 | 29 | <5 | 60 | 9 | 47 | 10 |
|  | R04 Haemorrhage from respiratory passages | 37 | 0 | 14 | <5 | 13 | <5 | 24 | 5 |
|  | R05 Cough | 35 | <5 | 29 | 0 | 55 | <5 | 21 | <5 |
|  | R06 Abnormalities of breathing | 160 | 34 | 57 | 9 | 142 | 13 | 56 | <5 |
|  | R07 Pain in throat and chest | 91 | 30 | 99 | 8 | 93 | 13 | 73 | 22 |
|  | R09 Other symptoms and signs involving the circulatory and respiratory systems | 8 | 0 | 0 | 0 | <5 | 0 | 0 | 0 |
| Digestive system and abdomen | R10 Abdominal and pelvic pain | 1277 | 132 | 445 | 59 | 1191 | 117 | 426 | 47 |
|  | R11 Nausea and vomiting | 97 | <5 | 97 | <5 | 107 | 9 | 59 | <5 |
|  | R13 Dysphagia | 17 | <5 | 30 | <5 | 38 | 0 | 14 | 0 |
| Skin and subcutaneous tissue | R20 Disturbances of skin sensation | 18 | 5 | 10 | <5 | 23 | <5 | 8 | 0 |
|  | R21 Rash and other nonspecific skin eruption | 33 | 8 | 20 | 7 | 30 | <5 | 21 | 5 |
|  | R22 Localized swelling, mass and lump of skin and subcutaneous tissue | 14 | <5 | 24 | <5 | 18 | <5 | 22 | 5 |
| Cognition, perception, emotional state, and behavior | R41.8 Other and unspecified symptoms and signs involving cognitive functions and awareness | 1052 | 64 | 928 | 132 | 628 | 67 | 648 | 62 |
|  | R42 Dizziness and giddiness | 82 | 10 | 46 | 6 | 92 | 8 | 36 | <5 |
|  | R43 Disturbances of smell and taste | 20 | 0 | <5 | 0 | 5 | 0 | 9 | <5 |
| General symptoms and signs | R50 Fever | 93 | 15 | 24 | <5 | 76 | <5 | 55 | <5 |
|  | R51Headache | 433 | 38 | 128 | 27 | 408 | 35 | 163 | 15 |
|  | R52 Pain | 116 | <5 | 84 | 12 | 171 | 11 | 83 | <5 |
|  | R53 Fatigue | 34 | <5 | 10 | <5 | 46 | <5 | <5 | <5 |
|  | R55 Syncope | 284 | 44 | 61 | <5 | 187 | 32 | 87 | 21 |
|  | R56 Convulsion | 29 | <5 | <5 | <5 | 43 | <5 | 24 | <5 |
|  | R59 Enlarged lymph nodes | 29 | <5 | 51 | <5 | <5 | <5 | <5 | <5 |
|  | **2. vaccine** |  | |  | |  | |  | |
|  | ***Short (0-56 days)*** |  | |  | |  | |  | |
| Circulatory and respiratory symptoms | R00 Abnormalities of heartbeat | 17 | 47 | <5 | <5 | 60 | <5 | 45 | 19 |
|  | R04 Haemorrhage from respiratory passages | <5 | <5 | <5 | <5 | <5 | <5 | <5 | <5 |
|  | R05 Cough | <5 | 36 | 22 | <5 | 55 | <5 | 19 | 12 |
|  | R06 Abnormalities of breathing | 94 | 23 | 31 | 31 | 140 | 37 | 54 | 26 |
|  | R07 Pain in throat and chest | 27 | 86 | 58 | 29 | 88 | 47 | 70 | 44 |
|  | R09 Other symptoms and signs involving the circulatory and respiratory systems | <5 | <5 | <5 | <5 | <5 | <5 | <5 | <5 |
| Digestive system and abdomen | R10 Abdominal and pelvic pain | 1344 | 381 | 370 | 202 | 2280 | 411 | 824 | 171 |
|  | R11 Nausea and vomiting | 22 | 46 | 66 | <5 | 113 | 34 | 55 | 19 |
|  | R13 Dysphagia | 7 | 6 | 19 | 13 | 43 | 9 | 14 | <5 |
| Skin and subcutaneous tissue | R20 Disturbances of skin sensation | 7 | <5 | <5 | 5 | 22 | 11 | 8 | <5 |
|  | R21 Rash and other nonspecific skin eruption | 25 | <5 | 16 | <5 | 30 | 6 | 20 | 6 |
|  | R22 Localized swelling, mass and lump of skin and subcutaneous tissue | 7 | <5 | 22 | 0 | 18 | <5 | 21 | 5 |
| Cognition, perception, emotional state, and behavior | R41.8 Other and unspecified symptoms and signs involving cognitive functions and awareness | 497 | 323 | 468 | 316 | 615 | 210 | 593 | 171 |
|  | R42 Dizziness and giddiness | 59 | <5 | 22 | <5 | 86 | 22 | 34 | <5 |
|  | R43 Disturbances of smell and taste | <5 | <5 | <5 | <5 | <5 | <5 | <5 | <5 |
| General symptoms and signs | R50 Fever | 33 | <5 | <5 | <5 | 78 | 21 | 55 | 29 |
|  | R51 Headache | 212 | 120 | 76 | 80 | 405 | 106 | 159 | 49 |
|  | R52 Pain | 61 | <5 | 46 | <5 | 159 | 43 | 79 | 19 |
|  | R53 Fatigue | 16 | <5 | <5 | <5 | 45 | 14 | <5 | <5 |
|  | R55 Syncope | 149 | 37 | 23 | <5 | 182 | 85 | 87 | 36 |
|  | R56 Convulsion | <5 | 21 | <5 | <5 | 43 | <5 | 24 | <5 |
|  | R59 Enlarged lymph nodes | <5 | <5 | 37 | <5 | <5 | <5 | <5 | 11 |
|  | ***Long (56-182 days)*** |  | |  | |  | |  | |
| Circulatory and respiratory symptoms | R00 Abnormalities of heartbeat | <5 | 62 | <5 | 40 | 87 | 56 | 67 | 41 |
|  | R04 Haemorrhage from respiratory passages | 20 | <5 | <5 | 23 | 19 | 19 | 36 | <5 |
|  | R05 Cough | <5 | 63 | <5 | 47 | 81 | 56 | 26 | 31 |
|  | R06 Abnormalities of breathing | 138 | 67 | <5 | <5 | 198 | 79 | 80 | 38 |
|  | R07 Pain in throat and chest | 37 | 250 | 36 | 157 | 128 | 88 | 94 | 72 |
|  | R09 Other symptoms and signs involving the circulatory and respiratory systems | <5 | <5 | <5 | <5 | <5 | <5 | <5 | <5 |
| Digestive system and abdomen | R10 Abdominal and pelvic pain | 788 | 1247 | 224 | 434 | 1632 | 759 | 611 | 286 |
|  | R11 Nausea and vomiting | 67 | 181 | 66 | 49 | 165 | 87 | 81 | 59 |
|  | R13 Dysphagia | 5 | <5 | 27 | <5 | 51 | 11 | 18 | 7 |
| Skin and subcutaneous tissue | R20 Disturbances of skin sensation | 7 | 16 | <5 | 0 | 27 | 13 | 11 | 9 |
|  | R21 Rash and other nonspecific skin eruption | 36 | 26 | 18 | 0 | 40 | 21 | 34 | 14 |
|  | R22 Localized swelling, mass and lump of skin and subcutaneous tissue | 7 | 49 | 15 | 0 | 27 | 11 | 24 | 9 |
| Cognition, perception, emotional state, and behavior | R41.8 Other and unspecified symptoms and signs involving cognitive functions and awareness | 396 | 560 | 524 | 992 | 760 | 569 | 842 | 417 |
|  | R42 Dizziness and giddiness | 64 | <5 | 14 | <5 | 132 | 59 | 55 | 15 |
|  | R43 Disturbances of smell and taste | <5 | <5 | <5 | <5 | <5 | <5 | <5 | <5 |
| General symptoms and signs | R50 Fever | 59 | 62 | <5 | 14 | 115 | 35 | 85 | 43 |
|  | R51 Headache | 345 | 282 | 82 | 274 | 587 | 229 | 247 | 120 |
|  | R52 Pain | 96 | <5 | 38 | <5 | 211 | 86 | 123 | 52 |
|  | R53 Fatigue | <5 | 107 | <5 | <5 | 67 | 24 | 18 | 41 |
|  | R55 Syncope | 150 | 225 | 29 | 115 | 275 | 148 | 122 | 87 |
|  | R56 Convulsion | <5 | <5 | <5 | <5 | 56 | 31 | 31 | <5 |
|  | R59 Enlarged lymph nodes | <5 | <5 | 53 | <5 | <5 | <5 | 11 | <5 |

A minimum of 5 events both before and after the index-date is a priori defined as required for the specific category to enter in the analyses. Due to Danish legislation events fewer than five cannot be shown.

^a^Number of events are reported 6 months prior to index-date and 0-21 days after the first vaccine dose and 0-56 days and 57-182 days after the second vaccine dose.
