# Supplementary material for: Symptom-Specific Hospital Contacts in 12–18-Year-Olds Vaccinated against COVID-19: A Danish Register-Based Cohort Study
Source: Vaccines (Basel). 2023 May 31;11(6):1049. doi: 10.3390/vaccines11061049 (PMC10301149; doi:10.3390/vaccines11061049)
Supplement: Supplementary file 1 [file vaccines-11-01049-s001.zip › vaccines-2391855-Supplementary.pdf]

Supplemental Information

Supplement to: Berg SK, Wallach-Kildemoes H, Rasmussen LR, et al. Symptom-specific hospital contacts in 12-18-year-olds vaccinated against COVID-19: A Danish register-based cohort study

Investigators 2

Supplemental Table S1. Selected ICD-10 R codes as outcomes. 3

Supplemental Table S2. Joint binary register-based measures for prevalent somatic and psychiatric disorders prior to first dose vaccine index-date, applying hospital diagnosis (ICD 10 codes) and eventually medicine proxies (ATC-codes) with specified look back period. 4

Supplemental Table S3. Characteristics of adolescents vaccinated against COVID-19 and matched unvaccinated: First dose during May-September and second dose 3-7 weeks later. 5

Supplemental Table S4. PERR and number of symptoms (ICD-10 R-codes) per 1000 person years at risk (rates) 6 months before and 21 days after the first vaccine dose (index-date) among vaccinated and matched unvaccinated adolescents aged 12-18 years. 6

Supplemental Table S5. Symptoms for first and second vaccine dose: Events 6 months before and three time periods after vaccination (index-date) among vaccinated and matched unvaccinated adolescents aged 12-18 years, stratified on age-groups. 7

Investigators

Selina Kikkenborg Berg [1,2,*], Helle Wallach-Kildemoes [1], Line Ryberg Rasmussen [1], Ulrikka Nygaard [2,3], Nina Marie Birk [1], Henning Bundgaard [1,2], Annette Kjær Ersbøll [4], Lau Caspar Thygesen [4], Susanne Dam Nielsen [2,5] and Anne Vinggaard Christensen [1]

[1] Department of Cardiology, Rigshospitalet, Copenhagen University Hospital, Inge Lehmanns Vej 7, 2100 Copenhagen, Denmark
[2] Faculty of Health and Medical Sciences, University of Copenhagen, Blegdamsvej 3B, 2200 Copenhagen, Denmark
[3] Department of Paediatrics and Adolescents Medicine, Rigshospitalet, Copenhagen University Hospital, Blegdamsvej 9, 2100 Copenhagen, Denmark
[4] National Institute of Public Health, University of Southern Denmark, Studiestræde 6, 1455 Copenhagen, Denmark
[5] Department of Infectious Disease, Rigshospitalet, Copenhagen University Hospital, Blegdamsvej 9, 2100 Copenhagen, Denmark
* Correspondence: selina@rh.dk

Supplemental Table S1. Selected ICD-10 R codes as outcomes.

R codes categories	Specific R codes
Circulatory and respiratory symptoms	R00 Abnormalities of heartbeat
	R04 Haemorrhage from respiratory passages
	R05 Cough
	R06 Abnormalities of breathing
	R07 Pain in throat and chest
	R09 Other symptoms and signs involving the circulatory and respiratory systems
Digestive system and abdomen	R10 Abdominal and pelvic pain
	R11 Nausea and vomiting
	R13 Dysphagia
Skin and subcutaneous tissue	R20 Disturbances of skin sensation
	R21 Rash and other nonspecific skin eruption
	R22 Localized swelling, mass and lump of skin and subcutaneous tissue
Cognition, perception, emotional state, and behavior	R41.8 Other and unspecified symptoms and signs involving cognitive functions and awareness
	R42 Dizziness and giddiness
	R43 Disturbances of smell and taste
General symptoms and signs	R50 Fever of other and unknown origin
	R51 Headache
	R52 Pain, not elsewhere classified
	R53 Malaise and fatigue
	R55 Syncope and collapse
	R56 Convulsions, not elsewhere classified
	R59 Enlarged lymph nodes

Supplemental Table S2. Joint binary register-based measures for prevalent somatic and psychiatric disorders prior to first dose vaccine index-date, applying hospital diagnosis (ICD 10 codes) and eventually medicine proxies (ATC-codes) with specified look back period.

Disorder categories	Register-based disorder defintions[a]: Prior diagnosis (ICD 10) / filled of prescription drug (ATC)	Look back period[b]
Asthma[c]	**ICD10**	
	J45-J46	Full
	**ATC**	
	Bronchodilating agents(comb): R03BA; R03AK; R03AL08; R03AL09; R03AL11-12; R03AC12- R03AC19; R03BB01;	Medium
	Leukotriene receptor antagonist: R03DC03	Medium
Other respiratory disorders[c]	**ICD10**	
	E84; J41-44; J47; J84; J96.1; P27;	Full
	J40	Short
Cardiovascular disorders[c]	**ICD10**	
	I05-I09; I27; I34-39; I41-I47; I50-I52	Full
	I01; I31; I33; I40	Medium
Renal disorders incl. dialysis[c]	**ICD10:**	
	N03; N04-5; N07-8; N18-19; N25-N27; Y84.1	Full
	N00-1; N17	Medium
Diabetes mellitus I or II[c]	**ICD10:**	
	E10-E11; Z96.4	Full
	**ATC:**	
	A10A	Medium
Endocrine disorders. excl. diabetes[c]	**ICD10**	
	E03.3; E03.5-9; E05-06; E27.1; E27.3-4	Full
	E27.2	Short
Hematologic disorders[c]	**ICD10**	
	D59.0-1; D69.0; D69.3; D86	Full
Dermatologic diseases[c]	**ICD10**	
	L10; L12; L13.0	Medium
	L20; L40; L63; L80	Full
Rheumatological disorders[c]	**ICD10**	
	M05-M09; M30-36; M45; M60 (excl. M60.0)	Full
Neuromuscular disorders[c]	**ICD10**	
	G35-37; G70-71; G80; G82	Full
	G61;	Medium
Gastrointestinal disorders incl. cirrhosis (any cause)[c]	**ICD10**	
	K50-51; K70.2-3; K71.7; K74; K76.1; K78.8; K900;	Full
	K35	Short
Congenital malformations and chromosomal abnormalities[c]	**ICD10**	
	Q20-28; Q31-34; Q90-95	Full
Malignancy[c]	**ICD10**	
	C00-97	Full
	**ATC**	
	L01	Full
Organ transplantation or immunodeficiency[c]	**ICD 10**	
	Organ transplantation: Z94 excl. Z94.5. Z94.7	Full
	Immunodeficiency: D70-72; D730; D80-84	Full
	**ATC**	
	L04	Full
Psychiatric disorders[d]	ICD10	
	F (any psychiatric primary diagnoses)	Full

[a]Individuals with a list of disorders within a look back period prior to survey completion date were identified, applying individual-level register information, corresponding to primary discharge diagnoses (ICD-10 codes) and/or prescription drug use disease proxies (ATC codes). The list includes somatic health conditions in adolescents conferring an increased risk of SARS-CoV-2 infection[46] along with any psychiatric discharge disorders.
[b]Looking back periods from the index-date: Full (ICD: full historic); Medium (ICD: 1 year; ATC: 6 months); Short (ICD: 3 months; ATC: 2 month); Very short: 14 days (ICD & ATC).
[c]A joint binary measure for prevalent somatic disorder: Yes (Yes in any of the listed prevalent disorders) No (No as to all included prevalent disorders).
[d]A joint measure for prevalent psychiatric disorder.

Supplemental Table S3. Characteristics of adolescents vaccinated against COVID-19 and matched unvaccinated: First dose during May-September and second dose 3-7 weeks later.

Category	First dose vaccine[a]		Second dose vaccine[b]			
	Vaccinated	Unvaccinated	Vaccinated	Unvaccinated according to follow-up		
				0-21 days	0-56 days	57-182 days
**All n (%)[c]**	(n=109,166)	(n=109,166)	(n=105,316)	(n=105,316)	(n=105,316)	(n=105,316)
**Unique individuals, n (%)[d]**	109,166 (100.0%)	27,106 (39.4%)	105,316 (100.0%)	25,415 (24.1%)	21,833 (20.7%)	14,007 (13.3%)
**Sex (Girl, %)**	53,423 (48.9%)	53,423 (48.9%)	51,665 (49.1%)	51,665 (49.1%)	51,665 (49.1%)	51,665 (49.1%)
**Age (mean, SD)**	15.6 (1.9)	15.4 (2.1)	15.5 (1.9)	15.4 (2.1)	15.4 (2.1)	15.4 (2.1)
**12 - 15 years (%)**	47,719 (43.7%)	47,719 (43.7%)	46,733 (44.4%)	46,733 (44.4%)	46,733 (44.4%)	46,733 (44.4%)
**16 - 18 years (%)**	61,447 (56.3%)	61,447 (56.3%)	58,583 (55.6%)	58,583 (55.6%)	58,583 (55.6%)	58,583 (55.6%)
**Prevalent health condition, n/yes (%)[e]**						
**Any of listed somatic diseases**	14,456 (13.2%)	12,961 (11.9%)	13,971 (13.3%)	12,220 (11.6%)	12,324 (11.7%)	12,197 (11.6%)
**Any registered psychiatric diagnosis**	8780 (8.0%)	10,054 (9.2%)	8377 (8.0%)	9966 (9.5%)	10,284 (9.8%)	10,468 (9.9%)
**Parental socio-economic position**						
**Highest formal parental education, n (%)**						
**Basic education**	5087 (4.7%)	13,098 (12.0%)	4737 (4.5%)	14,890 (14.1%)	15,230 (14.5%)	17,631 (16.7%)
**High school and vocational training**	44,777 (41.0%)	50,522 (46.3%)	43,245 (41.1%)	49,746 (47.2%)	50,777 (48.2%)	52,260 (49.6%)
**Higher education**	59,302 (54.3%)	45,546 (41.7%)	57,334 (54.4%)	40,680 (38.6%)	39,309 (37.3%)	35,425 (33.6%)
**Annual family income, n (%)**						
**Low (1. tertile)**	29,055 (26.6%)	50,078 (45.9%)	27,615 (26.2%)	53,858 (51.1%)	54,884 (52.1%)	59,961 (56.9%)
**Middle (2. tertile)**	34,172 (31.3%)	31,824 (29.2%)	33,168 (31.5%)	29,595 (28.1%)	29,734 (28.2%)	28,450 (27.0%)
**High (3. tertile)**	45,359 (41.6%)	26,296 (24.1%)	43,984 (41.8%)	20,857 (19.8%)	19,715 (18.7%)	15,901 (15.1%)
**Maternal citizenship, n (%)**						
**Danish**	101,063 (92.6%)	89,218 (81.7%)	97,754 (92.8%)	82,554 (78.4%)	82,037 (77.9%)	78,905 (74.9%)
**Other Western countries**	3261 (3.0%)	5194 (4.8%)	3089 (2.9%)	5685 (5.4%)	5922 (5.6%)	6778 (6.4%)
**Non-western countries**	4621 (4.2%)	14,160 (13.0%)	4277 (4.1%)	16,409 (15.6%)	16,684 (15.8%)	18,928 (18.0%)
**Unknown**	221 (0.2%)	594 (0.5%)	196 (0.2%)	668 (0.6%)	673 (0.6%)	705 (0.7%)

[a]Applying matching with replacement, unvaccinated were each week during the inclusion period (May-September 2021) matched to first dose vaccinated according to sex and age-group. Hence, unvaccinated may receive the vaccine during the inclusion period and be matched with an individual still unvaccinated. By the end of the inclusion period 27,058 unique individuals were still unvaccinated.
[b]Applying matching with replacement, unvaccinated were each week matched to second dose vaccinated according to sex and age-group. Merely individuals not receiving first dose vaccine during the respective follow-up periods were included.
[c]Matched number of index-date records applied in the analyses. Individuals still unvaccinated may have been assigned several index-dates. Index-date among vaccinated = date of vaccination. Prevalent health conditions and parental socio-economic position are based on these matched number of index-date records.
[d]Number unique individual applied for the matching. By the end of the inclusion period 27,058 individuals had not received first dose vaccine.
[e]Somatic or psychiatric disorders registered prior to first dose vaccine index-date: For details see Supplemental Table 2.

Supplemental Table S4. PERR[a] and number of symptoms (ICD-10 R-codes)[b] per 1000 person years at risk (rates) 6 months before and 21 days after the first vaccine dose (index-date) among vaccinated and matched unvaccinated adolescents aged 12-18 years.

	**Girls**						**Boys**					
	**Unvaccinated (Rate)**		**Vaccinated (Rate)**				**Unvaccinated (Rate)**		**Vaccinated (Rate)**			
	**before**	**after**	**before**	**after**	**PERR crude**	**PERR (95% CI)[c]**	**before**	**after**	**before**	**after**	**PERR crude**	**PERR (95% CI)[c]**
**1. Vaccine**												
(0-21 days)												
R00 Abnormalities of heartbeat	1.67	1.95	2.24	2.93	1.12	1.23 (0.34-#)	-	-	-	-	-	-
R06 Abnormalities of breathing	5.93	11.06	5.31	4.23	0.43	0.35 (0.14-1.26)	-	-	-	-	-	-
R07 Pain in throat and chest	3.37	9.76	3.48	4.23	0.42	0.46 (0.18-1.05)	3.53	2.49	2.62	6.86	3.71	4.70 (1.68-21.33)
R10 Abdominal and pelvic pain	47.41	42.95	44.54	38.08	0.94	0.91 (0.65-1.42)	15.84	18.40	15.27	14.66	0.83	0.90 (0.46-1.84)
R21 Rash and other nonspecific skin eruption	-	-	-	-	-	-	0.71	2.18	0.75	1.56	0.68	0.63 (0.11-4.08)
R41.8 Other and unspecified symptoms and signs involving cognitive functions and awareness	39.01	20.82	23.48	21.81	1.74	1.72 (1.10-2.96)	33.05	41.17	23.22	19.34	0.67	0.61 (0.32-1.45)
R42 Dizziness and giddiness	3.04	3.25	3.44	2.60	0.71	0.86 (0.11-#)	-	-	-	-	-	-

[a]PERR (Prior event rate ratio) = Ratio after (Vaccinated Rate after/Unvaccinated Rate after) divided by the Ratio before (Vaccinated Rate before/Unvaccinated Rate before).

[b]For the number of events for each symptom see Supplemental Table 5.

[v]By means of Poisson regression models, the ratio before and after is adjusted for time with SARS-CoV-2 infection in the follow-up period, highest attained parental education and age group (12-15 years and 16-18 years during inclusion period). 95% confidence interval (CI) of the PERR estimate through Bootstrapping with 200 replicates.

[#] Not possible to calculate the upper 95% CI (e.g., e+07).

Supplemental Table S5. Symptoms for first and second vaccine dose: Events 6 months before and three time periods[a] after vaccination (index-date) among vaccinated and matched unvaccinated adolescents aged 12-18 years, stratified on age-groups.

**Diagnoses by length of follow-up period**		**Non-vaccinated: Events (before/after)**				**Vaccinated: Events (before/after)**			
		**Girls**		**Boys**		**Girls**		**Boys**	
		**12-18**		**12-18**		**12-18**		**12-18**	
		before	*after*	*before*	*after*	*before*	*after*	*before*	*after*
	**1. vaccine**								
	Very-short (0-21 days)								
Circulatory and respiratory symptoms	R00 Abnormalities of heartbeat	45	6	29	<5	60	9	47	10
	R04 Haemorrhage from respiratory passages	37	0	14	<5	13	<5	24	5
	R05 Cough	35	<5	29	0	55	<5	21	<5
	R06 Abnormalities of breathing	160	34	57	9	142	13	56	<5
	R07 Pain in throat and chest	91	30	99	8	93	13	73	22
	R09 Other symptoms and signs involving the circulatory and respiratory systems	8	0	0	0	<5	0	0	0
Digestive system and abdomen	R10 Abdominal and pelvic pain	1277	132	445	59	1191	117	426	47
	R11 Nausea and vomiting	97	<5	97	<5	107	9	59	<5
	R13 Dysphagia	17	<5	30	<5	38	0	14	0
Skin and subcutaneous tissue	R20 Disturbances of skin sensation	18	5	10	<5	23	<5	8	0
	R21 Rash and other nonspecific skin eruption	33	8	20	7	30	<5	21	5
	R22 Localized swelling, mass and lump of skin and subcutaneous tissue	14	<5	24	<5	18	<5	22	5
Cognition, perception, emotional state, and behavior	R41.8 Other and unspecified symptoms and signs involving cognitive functions and awareness	1052	64	928	132	628	67	648	62
	R42 Dizziness and giddiness	82	10	46	6	92	8	36	<5
	R43 Disturbances of smell and taste	20	0	<5	0	5	0	9	<5
General symptoms and signs	R50 Fever	93	15	24	<5	76	<5	55	<5
	R51Headache	433	38	128	27	408	35	163	15
	R52 Pain	116	<5	84	12	171	11	83	<5
	R53 Fatigue	34	<5	10	<5	46	<5	<5	<5
	R55 Syncope	284	44	61	<5	187	32	87	21
	R56 Convulsion	29	<5	<5	<5	43	<5	24	<5
	R59 Enlarged lymph nodes	29	<5	51	<5	<5	<5	<5	<5
	**2. vaccine**								
	Short (0-56 days)								
Circulatory and respiratory symptoms	R00 Abnormalities of heartbeat	17	47	<5	<5	60	<5	45	19
	R04 Haemorrhage from respiratory passages	<5	<5	<5	<5	<5	<5	<5	<5
	R05 Cough	<5	36	22	<5	55	<5	19	12

	R06 Abnormalities of breathing	94	23	31	31	140	37	54	26
	R07 Pain in throat and chest	27	86	58	29	88	47	70	44
	R09 Other symptoms and signs involving the circulatory and respiratory systems	<5	<5	<5	<5	<5	<5	<5	<5
Digestive system and abdomen	R10 Abdominal and pelvic pain	1344	381	370	202	2280	411	824	171
	R11 Nausea and vomiting	22	46	66	<5	113	34	55	19
	R13 Dysphagia	7	6	19	13	43	9	14	<5
Skin and subcutaneous tissue	R20 Disturbances of skin sensation	7	<5	<5	5	22	11	8	<5
	R21 Rash and other nonspecific skin eruption	25	<5	16	<5	30	6	20	6
	R22 Localized swelling, mass and lump of skin and subcutaneous tissue	7	<5	22	0	18	<5	21	5
Cognition, perception, emotional state, and behavior	R41.8 Other and unspecified symptoms and signs involving cognitive functions and awareness	497	323	468	316	615	210	593	171
	R42 Dizziness and giddiness	59	<5	22	<5	86	22	34	<5
	R43 Disturbances of smell and taste	<5	<5	<5	<5	<5	<5	<5	<5
General symptoms and signs	R50 Fever	33	<5	<5	<5	78	21	55	29
	R51 Headache	212	120	76	80	405	106	159	49
	R52 Pain	61	<5	46	<5	159	43	79	19
	R53 Fatigue	16	<5	<5	<5	45	14	<5	<5
	R55 Syncope	149	37	23	<5	182	85	87	36
	R56 Convulsion	<5	21	<5	<5	43	<5	24	<5
	R59 Enlarged lymph nodes	<5	<5	37	<5	<5	<5	<5	11
	Long (56-182 days)								
Circulatory and respiratory symptoms	R00 Abnormalities of heartbeat	<5	62	<5	40	87	56	67	41
	R04 Haemorrhage from respiratory passages	20	<5	<5	23	19	19	36	<5
	R05 Cough	<5	63	<5	47	81	56	26	31
	R06 Abnormalities of breathing	138	67	<5	<5	198	79	80	38
	R07 Pain in throat and chest	37	250	36	157	128	88	94	72
	R09 Other symptoms and signs involving the circulatory and respiratory systems	<5	<5	<5	<5	<5	<5	<5	<5
Digestive system and abdomen	R10 Abdominal and pelvic pain	788	1247	224	434	1632	759	611	286
	R11 Nausea and vomiting	67	181	66	49	165	87	81	59
	R13 Dysphagia	5	<5	27	<5	51	11	18	7
Skin and subcutaneous tissue	R20 Disturbances of skin sensation	7	16	<5	0	27	13	11	9
	R21 Rash and other nonspecific skin eruption	36	26	18	0	40	21	34	14
	R22 Localized swelling, mass and lump of skin and subcutaneous tissue	7	49	15	0	27	11	24	9
Cognition, perception, emotional state, and behavior	R41.8 Other and unspecified symptoms and signs involving cognitive functions and awareness	396	560	524	992	760	569	842	417
	R42 Dizziness and giddiness	64	<5	14	<5	132	59	55	15
	R43 Disturbances of smell and taste	<5	<5	<5	<5	<5	<5	<5	<5

General symptoms and signs	R50 Fever	59	62	<5	14	115	35	85	43
	R51 Headache	345	282	82	274	587	229	247	120
	R52 Pain	96	<5	38	<5	211	86	123	52
	R53 Fatigue	<5	107	<5	<5	67	24	18	41
	R55 Syncope	150	225	29	115	275	148	122	87
	R56 Convulsion	<5	<5	<5	<5	56	31	31	<5
	R59 Enlarged lymph nodes	<5	<5	53	<5	<5	<5	11	<5

A minimum of 5 events both before and after the index-date is a priori defined as required for the specific category to enter in the analyses. Due to Danish legislation events fewer than five cannot be shown.

[a]Number of events are reported 6 months prior to index-date and 0-21 days after the first vaccine dose and 0-56 days and 57-182 days after the second vaccine dose.
